# Photovoltaic Array Fault Diagnosis Based on Gaussian Kernel Fuzzy C-Means Clustering Algorithm

**DOI:** 10.3390/s19071520

**Published:** 2019-03-28

**Authors:** Shengyang Liu, Lei Dong, Xiaozhong Liao, Xiaodong Cao, Xiaoxiao Wang

**Affiliations:** School of Automation, Beijing Institute of Technology, Beijing 100081, China; 3120140375@bit.edu.cn (S.L.); liaoxiaozhong@bit.edu.cn (X.L.); 2120160861@bit.edu.cn (X.C.); 2120160877@bit.edu.cn (X.W.)

**Keywords:** solar energy, PV array, fault diagnosis, fill factor, kernel fuzzy C-means Clustering, KFCM

## Abstract

In the fault diagnosis process of a photovoltaic (PV) array, it is difficult to discriminate single faults and compound faults with similar signatures. Furthermore, the data collected in the actual field experiment also contains strong noise, which leads to the decline of diagnostic accuracy. In order to solve these problems, a new eigenvector composed of the normalized PV voltage, the normalized PV current and the fill factor is constructed and proposed to characterize the common faults, such as open circuit, short circuit and compound faults in the PV array. The combination of these three feature characteristics can reduce the interference of external meteorological conditions in the fault identification. In order to obtain the new eigenvectors, a multi-sensory system for fault diagnosis in a PV array, combined with a data-mining solution for the classification of the operational state of the PV array, is needed. The selected sensors are temperature sensors, irradiance sensors, voltage sensors and current sensors. Taking account of the complexity of the fault data in the PV array, the Kernel Fuzzy C-means clustering method is adopted to identify these fault types. Gaussian Kernel Fuzzy C-means clustering method (GKFCM) shows good clustering performance for classifying the complex datasets, thus the classification accuracy can be effectively improved in the recognition process. This algorithm is divided into the training and testing phases. In the training phase, the feature vectors of 8 different fault types are clustered to obtain the training core points. According to the minimum Euclidean Distances between the training core points and new fault data, the new fault datasets can be identified into the corresponding classes in the fault classification stage. This strategy can not only diagnose single faults, but also identify compound fault conditions. Finally, the simulation and field experiment demonstrated that the algorithm can effectively diagnose the 8 common faults in photovoltaic arrays.

## 1. Introduction

With the intensification of the energy and environment crisis, clean energy plays an integral role in restraining global warming issues and has received more and more attention in industrial circles. As a major clean energy technology, photovoltaic power generation has received worldwide attention in recent years, especially in developing countries. Online intelligent multisensor monitoring systems are the guarantee of stable operation for the PV system. This direction is increasingly becoming a research hotspot in academic circles. Once the signal from the sensors have been acquired, various diagnostic techniques of the PV array can be adopted to extract as much information as possible from these signals. Furthermore, suitable decision-making strategies can be built up for failure detection of a PV array. A variety of studies have applied different diagnostic approaches to conduct this industrial task. In paper [1,2], Takashima T et al. used the acquired voltage and current signals to propose time domain reflectometry (TDR) and earth capacitance measurement (ECM) to detect the positions of the open circuit points in the circuits. Hu Y et al. presented a Thermography-based temperature distribution analysis method to identify PV module mismatch faults [3]. Nuri Gokmen et al. only measured the operating voltage of PV string and ambient temperature to efficiently detect the number of open and short circuit faults and discriminate between them and partial shading conditions [4]. Siva Ramakrishna Madeti, S.N. Singh proposed a program that using minimum number of voltage sensors to locate fault modules. The main advantage of this algorithm is that it voids the need of current sensors and reduces the number of the sensors used [5]. A. Chouder and S. Slivestre defined two new power loss indicators, including thermal capture losses and miscellaneous capture losses, to classify different fault types in PV system operations [6]. The line-to-line (LLF), ground fault (GF) and arc fault (AF) are generally considered to be the main catastrophic failures in a PV array [7,8,9]. Arc faults cause continuous oscillations and distortions in the output current and voltage waveforms of the inverter. Then the diagnosis of the fault type replies on a suitable time-frequency domain analysis on the voltage and current waveforms [10,11,12]. Fault detection in a PV array becomes difficult under low irradiance conditions. Line-to-line faults and ground faults may be undetected in the low irradiance situation. Artificial intelligence (AI) techniques are considered to be promising strategies to address with these problems. Zhehan Yi and Amir H. Etemadi proposed a multi-resolution signal decomposition method to extract fault features and two intelligent classification methods to identify the line-to-line and ground faults [13,14]. In addition, there are some other AI techniques for detecting the faults in a PV array. The strategy based on the fault threshold and Back Propagation (BP) neural networks was proposed in the paper [15] to detect 8 fault types at three levels including component, branch and array. In the literature [16], a multi-class support vector machine was designed to diagnose the line-to-line faults and the aging faults in the photovoltaic array. Zhao Y et al. [17] developed a graph-based semi-supervised detection method for discriminating different faults including short circuits, open circuits and line-to-line faults, and so on. The paper [18] proposed a density peak-based clustering approach for fault diagnosis in PV arrays. However, this approach needs the predetermination of the clustering number. Bi Rui et al. [19] combined the open circuit voltage *V*_oc_, short circuit current *I*_sc_, maximum power voltage *V*_mpp_ and maximum power current *I*_mpp_ as the feature vectors to characterize different fault types, then the Fuzzy C-means clustering method (FCM) was used to classify these faults.

Under the operating conditions, some single faults and compound faults display similar features and the fault datasets acquired in the field experiment are usually accompanied by environmental and system noise. These two points lead to the fault classification difficulty that some single faults and compound faults with similar characteristics cannot be discriminated by the conventional feature quantities proposed in previous papers. The conventional feature quantities mainly contain voltage, current, power, *V*_norm_, *I*_norm_, *FF* (*V*_norm_, *I*_norm_), and so on. In order to realize a good degree of fault discrimination, a new eigenvector composing of *V*_norm_, *I*_norm_ and *FF* was constructed to characterize 8 different faults in three-dimension space. The simulation results show that the proposed feature vectors can separate these faults effectively in noise-free conditions. As a result of the noise existing in the data collecting process of the field experiment, the new feature vectors cannot distinguish the 8 faults in the operating condition obviously. The Gaussian Kernel Fuzzy C-means clustering method (GKFCM) is an unsupervised learning clustering algorithm that using kernel function to map the sample data in the original space to a high-dimension space and then adopting the similarity function to classify the fault datasets. Compared to the traditional fuzzy C-means clustering algorithm, the GKFCM can highlight the difference of the sample characteristics by the nonlinear mapping of kernel space. This method can improve the clustering ability of complex datasets and robustness of fault diagnosis effectively. Therefore, a new fault classification method based on the GKFCM with the new eigenvectors (*V*_norm_, *I*_norm_, *FF*) is propounded in this paper. In the first phase of the algorithm, the different faults are characterized by the new eigenvectors and achieves a preliminary distinction. Then, the different fault datasets are input into the GKFCM to train and test for drawing fault diagnostic conclusions. Since the proposed method considers the normalization of the feature quantities and the robustness of the GKFCM, the generalization ability of the fault diagnostic algorithm can be further guaranteed.

## 2. Gaussian Kernel Fuzzy C-Means Clustering Method

The gaussian kernel fuzzy C-means clustering method contains two steps: firstly, the original space *X* is mapped to high-dimension space *F* by the Gaussian Kernel Function and then using the clustering algorithm to classify different datasets. The GKFCM can highlight the difference of the sample features by the non-linear mapping operation in the kernel space. This method is suitable for the classification problems with similar sample characteristics [20,21,22,23,24,25,26]. The nonlinear mapping function is defined as follow:

(1) $$\Phi :x_{k}\to \Phi \left(x_{k}\right)\in F$$

where $x_{k}$ is the sample of the original space *X*. The objective function of the clustering algorithm is given by

(2) $$J_{m}\left(U,\nu \right)=\sum_{i=1}^{c}\sum_{k=1}^{n}\mu_{ik}^{m}{\left\|\Phi \left(x_{k}\right)-\Phi \left(\nu_{i}\right)\right\|}^{2}$$

where $\nu_{i}$ is the core point of the original sample space; c is the clustering number; n is the sample number in the original sample space; $\mu_{ik}$ is the membership between the kth sample and the ith fault class. $\mu_{ik}$ satisfies three conditions including $\mu_{ik}\in \left[0,1\right]$, $0<\sum_{k=1}^{n}\mu_{ik}<n$ and $\sum_{i=1}^{c}\mu_{ik}=1,\;k=1,2,\cdots ,n$; m is a weighted parameter.

The kernel function is defined as follows:

(3) $$K\left(x,y\right)=\Phi {\left(x\right)}^{T}\Phi \left(y\right)$$

Therefore, the Euclidean distance in Equation (2) is given by

(4) $${\left\|\Phi \left(x_{k}\right)-\Phi \left(\nu_{i}\right)\right\|}^{2}=K\left(x_{k},x_{k}\right)+K\left(\nu_{i},\nu_{i}\right)-2K\left(x_{k},\nu_{i}\right)$$

The common kernel functions generally contain the Gaussian kernel function, polynomial kernel function and Sigmoid kernel function. The Gaussian kernel function is adopted in this paper and shown as the Equation (5).

(5) $$K\left(x_{k},\nu_{i}\right)=\exp\left[-{\left\|x_{k}-\nu_{i}\right\|}^{2}/\left(2\sigma^{2}\right)\right]$$

where σ represents the parameter of Gaussian kernel function. 

Altogether, the Equation (2) uses the constraints and the Lagrange multiplier approach to find $\mu_{ik}$ and $\nu_{i}$. The parameters $\mu_{ik}$ and $\nu_{i}$ are shown as the Equations (6) and (7).

(6) $$\mu_{ik}=\frac{{\left[1/\left(1-K\left(x_{k},\nu_{i}\right)\right)\right]}^{1/\left(m-1\right)}}{\sum_{j=1}^{c}{\left[1/\left(1-K\left(x_{k},\nu_{j}\right)\right)\right]}^{1/\left(m-1\right)}}$$

(7) $$\nu_{i}=\frac{\sum_{k=1}^{n}\mu_{ik}^{m}K\left(x_{k},\nu_{i}\right)x_{k}}{\sum_{k=1}^{n}\mu_{ik}^{m}K\left(x_{k},\nu_{i}\right)}$$

## 3. The Fault Diagnosis Algorithm Based on GKFCM

The fault diagnosis method based on the GKFCM is divided into two stages: the training and testing phases. In the training stage, using the training fault datasets to train the GKFCM and taking the fault classification error as the principle of the training performance. When the classification error reaches the minimum level, the core points are calculated and obtained. The classification error equation is shown by

(8) $$W=1-\frac{1}{c}\sum_{i=1}^{c}\frac{\left|C_{i}^{}\right|}{\left|C_{i}^{L}\right|}$$

where $C_{i}^{L}$ and $\left|C_{i}^{L}\right|$ are the ith class fault dataset and its corresponding sample number, respectively. c is the sample number of the fault dataset. $\left|C_{i}^{}\right|$ belongs to the ith fault class after the GKFCM clustering.

In the testing phase, the similarity between the new fault data and the center points of the reference fault datasets is used to judge the fault category of the new fault data. The steps of the fault diagnosis are listed as follows:

- Using the fault datasets including c classes as the training datasets
- According to the Equation (7), the core points of the reference datasets are calculated and obtained when the classification error rate reaches the minimum level.
- Using the Equations (9) and (10) to determine the fault type of the new sample $x_{\mathrm{new}}$ in the testing datasets.
  (9) $$\rho_{i}=\frac{2}{1+\exp\left(d_{h}\left(x_{\mathrm{new}},o_{i}\right)\right)}$$
  (10) $$\{\begin{array}{c} \underset{i=1,2,\cdots \cdot \cdot \cdot ,c}{\max\left\{\rho_{i}\right\}}\geq \lambda ,\;x_{\mathrm{new}}\in known\;fault\\ \underset{i=1,2,\cdots \cdot \cdot \cdot ,c}{\max\left\{\rho_{i}\right\}}<\lambda ,\;x_{\mathrm{new}}\in unknown\;fault \end{array}$$
  where $\rho_{i}$ is the similarity between $x_{\mathrm{new}}$ and the dataset $C_{i}^{L}$. The larger the value of $\rho_{i}$, the higher the possibility of $x_{\mathrm{new}}$ belonging to the corresponding fault type [15]. The Equation (8) is the judging criteria for the classification of the datasets in Support Vector Machine (SVM) and we use this principle to discriminate different fault datasets in this paper. λ is the category attribution threshold and the value is generally distributed between 0 and 0.5 [15,16].
- The previous step can not only determine the known faults in the training datasets but also judge the unknown fault types. If the $x_{\mathrm{new}}$ belongs to an unknown fault, it will be classified as the c+1th class.

**Figure 1:**
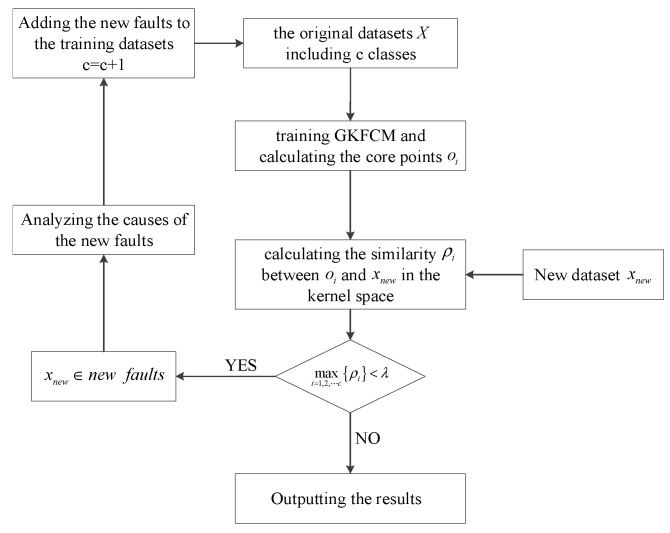
The flow chart of the fault diagnostic method based on Gaussian Kernel Fuzzy C-means clustering method (GKFCM).

The flow chart of the proposed fault diagnostic method are described as Figure 1.

## 4. The Fault Diagnosis Method Based on GKFCM for the Photovoltaic Arrays

### 4.1. Selection of the Fault Feature Quantities

In order to obtain a good diagnostic accuracy, three characteristics are constructed and combined as the feature vector for characterizing different fault types of PV array [16,17,18,27].

- The normalized PV voltage *V*_norm_
  (11) $$V_{\mathrm{norm}}=\frac{V_{\mathrm{mpp}}}{m\times V_{\mathrm{oc}-\mathrm{ref}}}$$
  where $V_{\mathrm{oc}-\mathrm{ref}}$ is the open circuit voltage of the reference PV module. m is the module number in each branch.
- The normalized PV current *I*_norm_
  (12) $$I_{\mathrm{norm}}=\frac{I_{\mathrm{mpp}}}{n\times I_{\mathrm{sc}-\mathrm{ref}}}$$
  where $I_{\mathrm{sc}-\mathrm{ref}}$ is the short circuit current of the reference PV module. n is the branch number of the PV array.
- The Fill Factor (*FF*)
  (13) $$FF=\frac{V_{m}I_{m}}{V_{\mathrm{oc}-\mathrm{array}}I_{\mathrm{sc}-\mathrm{array}}}$$
  where $V_{\mathrm{oc}-\mathrm{array}}$ is the open circuit voltage of the PV array and $I_{\mathrm{sc}-\mathrm{array}}$ is the short circuit current of the PV array.

The 4×3 PV array is designed as the research object in this paper and the detailed operating conditions contains four categories: normal, open circuit, short circuit and compound fault conditions. There are 8 fault types in total. c is the number of the fault types and equals to 8. The 8 fault types of the photovoltaic array are simulated in the simulation and the fault feature vectors X that characterizing the 8 faults are acquired and shown as the Equation (14).

(14) $$X=\left[\begin{array}{c} V_{\mathrm{norm}}{}^{1},I_{\mathrm{norm}}{}^{1},FF^{1}\\ \vdots \\ V_{\mathrm{norm}}{}^{i},I_{\mathrm{norm}}{}^{i},FF^{i}\\ \vdots \\ V_{\mathrm{norm}}{}^{n},I_{\mathrm{norm}}{}^{n},FF^{n} \end{array}\right]$$

where *X* is the datasets of 8 fault types. n is the sample number of each fault.

### 4.2. Procedures of PV Array Fault Detection Approach Based on GKFCM

- Firstly, using the acquired datasets of 8 fault types to train the GKFCM. The core points $o_{i},\;i=1,2,\ldots ,8$ are obtained when the classification error reaches the minimum level.
- Secondly, substituting the new fault dataset $x_{\mathrm{new}}$ into the trained GKFCM and using the Equations (9) and (10) to judge the fault types of the new fault data $x_{\mathrm{new}}$.
- Lastly, judging the fault type based on the maximum similarity between the center points of the reference fault datasets and the new fault $x_{\mathrm{new}}$. If $x_{\mathrm{new}}$ is not the known fault types included in the reference fault datasets, a new procedure is conducted to identify the fault type of $x_{\mathrm{new}}$. Then using it as a new fault type to train GKFCM to get a new training model.

## 5. The Simulation Experiment

### 5.1. Model of the Photovoltaic Array

The single diode model (SDM) shown as Equation (15) is the most commonly utilized model [28,29,30]. It is used to construct the 4×3 PV array to mimic accurately solar cells and PV modules behaviors in this paper.

(15) $$I=I_{\mathrm{ph}}-I_{\mathrm{sat}}\left(\exp\left(\frac{V+R_{s}I}{n\cdot V_{t}}\right)-1\right)-\left(\frac{V+R_{s}I}{R_{\mathrm{sh}}}\right)$$

The parameters of the PV module in the simulation are listed in Table 1.

**Figure 2:**
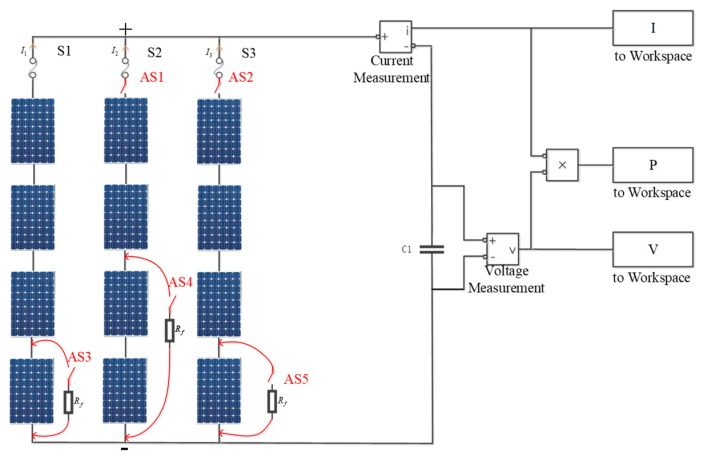
The simulation model of 4×3 PV array.

The simulation model of the 4×3 PV array is shown in Figure 2.

The wide-weather environment conditions in simulation experiment are set as the ranges of solar irradiance varying from 100 to 1000 W/m^2^ with step of 50 W/m^2^ and the backplane temperature changing from 0 °C to 40 °C with step of 1 °C. The 8 fault types can be simulated and carried out by the different combinations of Air Switches (AS) shown in Figure 2. The detailed descriptions of 8 fault types are listed in Table 2.

In the simulation experiment, 779 samples were acquired in each fault type of the 8 faults and 6332 fault samples were obtained in total.

### 5.2. The Feature Characteristic Analysis of 8 Fault Types

**Figure 3:**
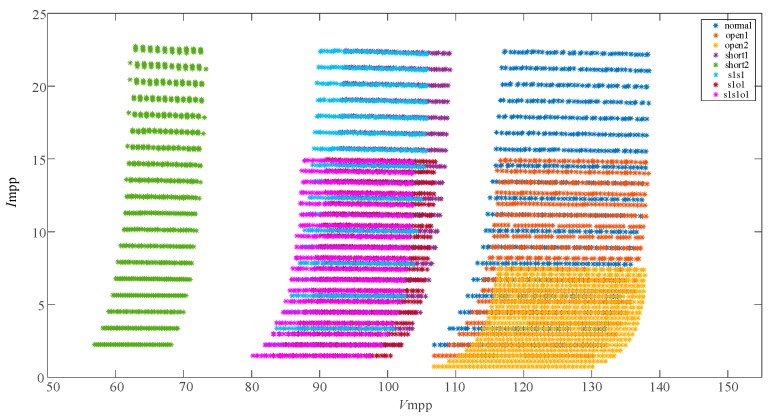
The distribution of the 8 fault characteristic points $\left(V_{\mathrm{mpp}},I_{\mathrm{mpp}}\right)$.

The conventional feature vectors $\left(V_{\mathrm{mpp}},I_{\mathrm{mpp}}\right)$ were used to characterize the 8 fault types in the paper [6] and the distribution of the eigenvectors $\left(V_{\mathrm{mpp}},I_{\mathrm{mpp}}\right)$ are shown in Figure 3. It is obvious that the 8 fault types overlap seriously and they are difficult to differentiate. 

**Figure 4:**
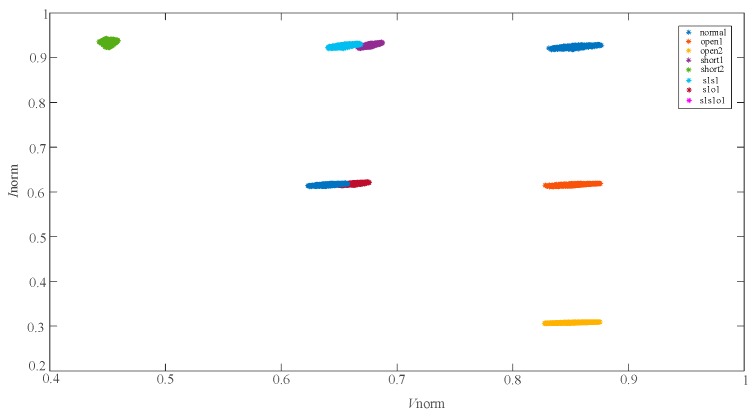
The distribution of the 8 fault characteristic points $\left(V_{\mathrm{norm}},I_{\mathrm{norm}}\right)$.

**Figure 5:**
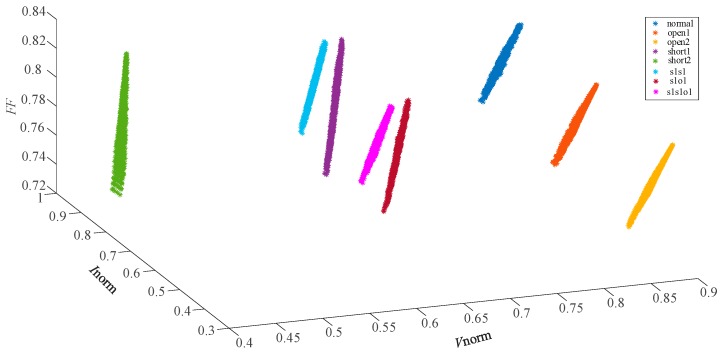
The distribution of the 8 faults characteristic points $\left(V_{\mathrm{norm}},I_{\mathrm{norm}},FF\right)$.

In the paper [6,7], the eigenvectors of 8 fault types are tranformed and improved as $\left(V_{\mathrm{norm}},I_{\mathrm{norm}}\right)$ for further discriminating the overlapping faults. The distribution of the feature vectors $\left(V_{\mathrm{norm}},I_{\mathrm{norm}}\right)$ are described in Figure 4 and the faults are mostly separated. However, short1 and s1s1, s1o1 and s1s1o1 still overlap with each other. The data distribution in Figure 3; Figure 4 indicates that the eigenvectors $\left(V_{\mathrm{mpp}},I_{\mathrm{mpp}}\right)$ and $\left(V_{\mathrm{norm}},I_{\mathrm{norm}}\right)$ have limitations in differentiating the single faults and compound faults with similar characteristics. It was found that the Filling Factor (*FF*) of each fault case was different through the in-depth research. So, the normalized PV voltage *V*_norm_, the normalized PV current *I*_norm_ and the Fill Factor (*FF*) were combined together as a new eigenvector to further characterize 8 different fault types. The results, showing use of the feature vectors $\left(V_{\mathrm{norm}},I_{\mathrm{norm}},FF\right)$ for fault characterization of the 8 faults, are shown in Figure 5.

The distribution of the eigenvectors $\left(V_{\mathrm{norm}},I_{\mathrm{norm}},FF\right)$ in Figure 5 demonstrates that the newly proposed feature vector can discriminate the 8 fault types obviously. This new eigenvector is appropriate for describing and characterizing some complex operating conditions, including single and compound faults with similar characteristics.

### 5.3. Simulation Results

Figure 5 gives an intuitive display that 8 faults can be separated by the eigenvectors $\left(V_{\mathrm{norm}},I_{\mathrm{norm}},FF\right)$. However, the actual data acquisition process is usually accompanied by the noise of the environment and acquisition devices. Therefore, the fault datasets acquired are complex and the differentiation degree of each fault is not obvious. Since GKFCM can effectively improve the clustering performance of the complex datasets, this clustering algorithm was adopted to cluster and identify fault types in this article. The classification and identification of the new fault datasets is based on the maximum similarity between the new fault datasets and the core points of the reference datasets. As the distribution of the 8 fault types is relatively concentrated in Figure 5, a small part of the fault datasets in wide-weather environmental conditions can be used as the reference datasets to train the GKFCM and then identify the new fault datasets. The environmental parameters in the 8 fault datasets collection process are listed in Table 3.

In the training phase of the simulation, the detailed parameters of GKFCM were set as follows: the clustering number was 8, weighted index m was 2, the maximum number of the iterations was 1000 and the limitation of the iteration was the minimum similarity with the value 10^−5^.

**Figure 6:**
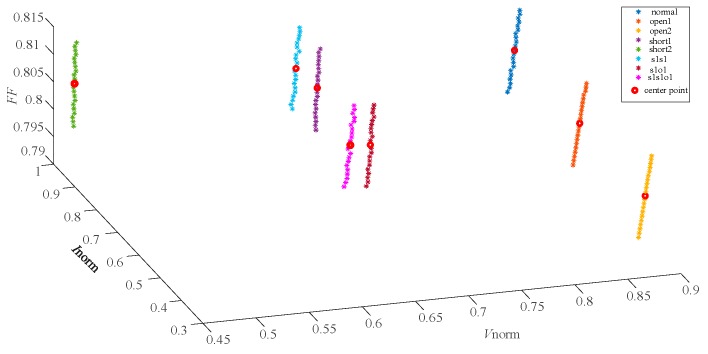
The training datasets and clustering center points of 8 faults.

The Figure 6 shows the distribution of the 8 faults in the simulated training datasets and the red points are the cores of each clustering. The center-point coordinates of the 8 clusterings in the reference fault datasets are listed in Table 4.

In the testing stage, each dataset of 8 fault types contained 110 samples. The Equations (9) and (10) were used to calculate the similarity of the new fault datasets and the core points $o_{i},\;i=1,2,\ldots ,8$ in the reference fault datasets. λ is set as 0.5 and the results of the classification are listed in Table 5.

In summary, the overall diagnostic accuracy was 100%. The simulation experiment shows that the proposed method has a good fault diagnostic performance. 

## 6. The Field Experiment

**Figure 7:**
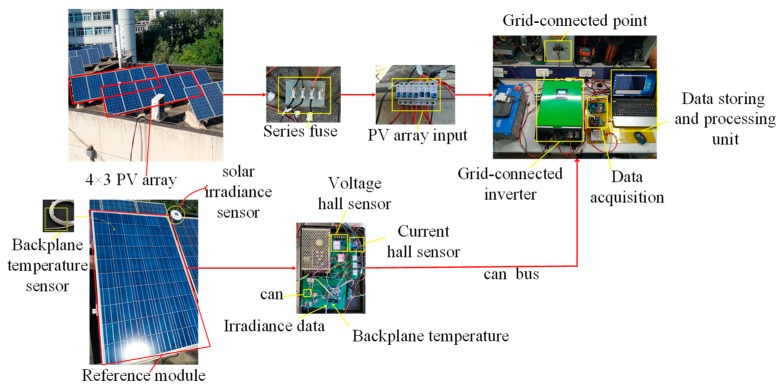
The platform of 4×3 PV array.

In the field experiment, the GSP-240 PV module was used to build the 4×3 PV array for validating the proposed fault diagnostic method. The key specifications of GSP-240 module are listed in Table 6 and the experiment platform is shown in Figure 7. The field experiment platform contained 4×3 PV array, 5 KW inverter, a series fuse, a combiner box and the data collecting and recording system. The data acquisition system consists of a temperature sensor, a solar irradiance sensor and two pairs of current and voltage sensors. The first two sensors and one pair of current and voltage sensors were located on the reference module. They were used to collect meteorological information, the short circuit current $I_{sc-ref}$ and open circuit voltage $V_{oc-ref}$ of the reference module, respectively. The operating voltage $V_{mpp}$ and current signals $I_{mpp}$ of PV array were measured by means of the other pair of the current and voltage sensors. This set of sensors shown in the data acquisition board in Figure 7 were mounted at the input of the converter. In total, a 6-sensor system was developed for online monitoring of the PV array. In this experiment, eight fault datasets were simulated by different combination of the air breakers and the different fault datasets were collected from 09:30 to 10:30 on 7 September 2018. In the fault datasets, each fault dataset owns 180 samples. Therein, 60 fault samples of each class were defined as the training datasets and the remaining 120 samples in each fault type were divided as the testing datasets. 

**Figure 8:**
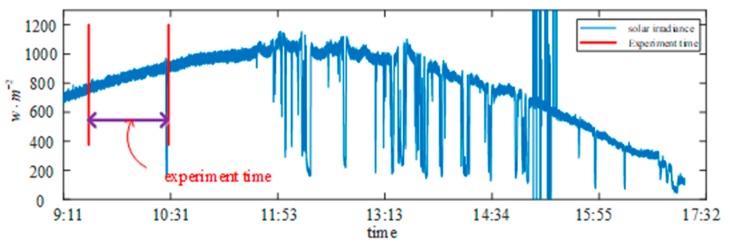

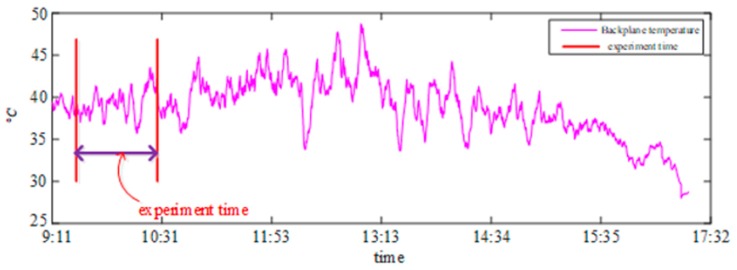
The solar irradiance and temperature range in the whole day on 7 September 2018.

The range of the irradiance and backplane temperature in the daytime on 7 September 2018 are shown in Figure 8.

**Figure 9:**
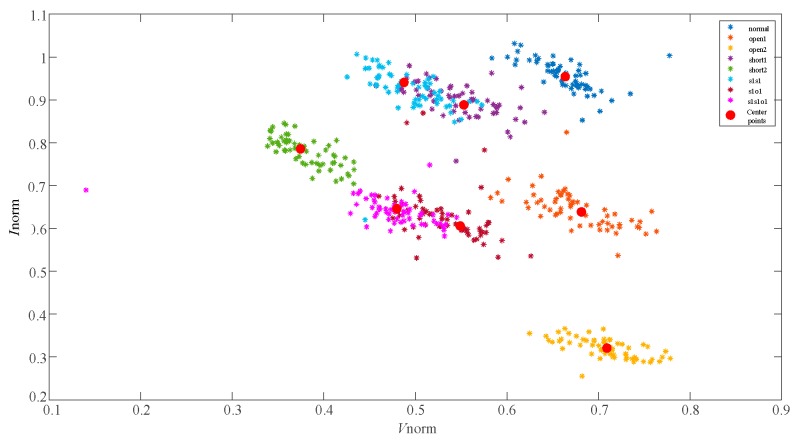
The distribution of eight fault characteristic points $\left(V_{\mathrm{norm}},I_{\mathrm{norm}}\right)$ in the field experiment.

**Figure 10:**
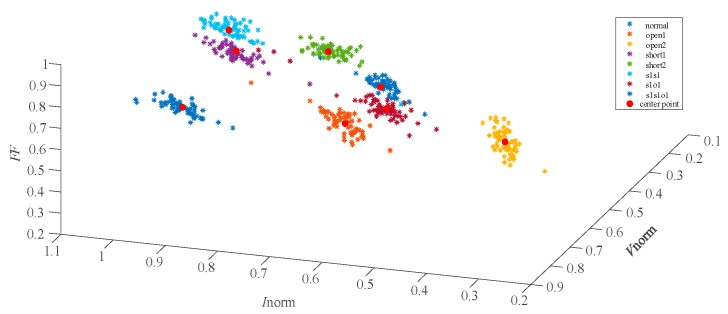
The distribution of eight fault characteristic points $\left(V_{\mathrm{norm}},I_{\mathrm{norm}},FF\right)$ and clustering center points in the field experiment.

The eigenvector $\left(V_{\mathrm{norm}},I_{\mathrm{norm}}\right)$ distribution of 8 fault types shown in Figure 9 indicates that some single faults and compound faults with similar characteristics are difficult to be discriminated in two-dimension space $\left(V_{\mathrm{norm}},I_{\mathrm{norm}}\right)$. In contrast, the feature vectors $\left(V_{\mathrm{norm}},I_{\mathrm{norm}},FF\right)$ that are shown distributed in Figure 10 can yield a more obvious discrimination of 8 fault types than the eigenvectors $\left(V_{\mathrm{norm}},I_{\mathrm{norm}}\right)$. After the training process of GKFCM, the kernels of the 8 fault training datasets with two-dimension and three-dimension feature characteristics were calculated and distributed as the red points in Figure 9 and Figure 10, respectively. 

In the fault identification phase of GKFCM, 120 samples of each fault are used to validate the effectiveness of the algorithm. The Equation (8) was used to calculate the similarity between the new fault data $x_{\mathrm{new}}$ and the core points $o_{i},\;i=1,2,\ldots ,8$ of the reference fault datasets. λ was set as 0.5. The results of fault diagnosis are listed in Table 7; Table 8, respectively. The Table 7 is the results acquired by processing two-dimension datasets and the Table 8 are the diagnostic performance that representing the processing results of the three-dimension fault datasets. In two tables, the first 8 faults in $x_{\mathrm{new}}$ belong to the corresponding categories. In addition, line to line fault was defined as an unknown fault for validating the ability of the algorithm to identify an unknown faults. The diagnostic results in the last row of Table 7 and Table 8 illustrate that the proposed method based on GKFCM can also identify the unknown fault accurately.

It can be seen in Table 7 and Table 8 that all 8 fault types within the training datasets and the unknown faults realize fine discrimination. The results of this experiment demonstrate that the proposed algorithm has a good diagnostic performance for 8 common faults, including 5 single faults and 3 compound faults in PV array.

**Figure 11:**
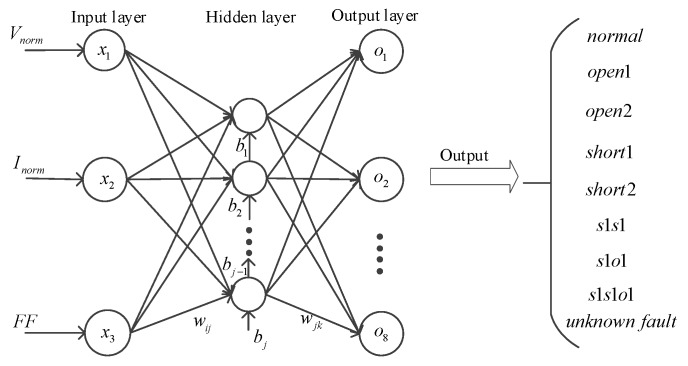
Basic structure of the BP neural network.

In order to further validate the persuasive and reliability of the proposed clustering method, a three-layer BP neural network was built to detect the 8 faults and an unknown fault. The structure of the BP neural network is shown in Figure 11. The BP neural network was trained by using the training datasets including 8 fault types, then the remaining fault samples of 8 fault types and the unknown fault samples were diagnosed by the trained BP neural network in the testing phase. The specific diagnostic results respectively characterized by two-dimension and three-dimension eigenvectors are shown in Table 9 and Table 10. The parameters in the BP neural network were set as follows: the iteration number was 1000, the learning rate was set as 0.01 and the minimum error was 0.01.

In the testing stage, two-dimension eigenvectors $\left(V_{\mathrm{norm}},I_{\mathrm{norm}}\right)$ were used to characterize the 8 fault types and the unknown faults. The corresponding results are shown in Table 9.

Meanwhile, three-dimension eigenvectors $\left(V_{\mathrm{norm}},I_{\mathrm{norm}},FF\right)$ were used to characterize the 8 fault types and the unknown faults. The final results are shown in Table 10.

In comparation with the results shown in Table 8 and Table 10, the processing algorithm with the two-dimension eigenvector has a lower diagnostic accuracy, and the details are shown in Table 7 and Table 9. These results demonstrate that the three-dimensional feature vectors, including the extended 3rd-dimension feature quantity, do improve the diagnostic accuracy of the faults. 

In addition, compared to the results shown in Table 7 and Table 8, the performance of the BP neural network shown in Table 9 and Table 10 for the 8 known faults was similar to the results acquired by the proposed algorithm. However, the unknown faults are all identified as normal when the unknown faults occur. In respect to the detection of the unknown faults, the BP neural network has poor generalization ability. In contrast, the detection method based on GKFCM still has a relatively better diagnostic result when the unknown faults occur. 

Overall, the results of the simulation and field experiments demonstrate that the strategy combining the new three-dimension eigenvectors and GKFCM proposed in this paper does not only exhibit a good diagnostic accuracy on the existing 8 fault types in the training datasets, but also can identify the unknown faults well. The algorithm presented has a good diagnostic accuracy and generalization capability.

## 7. Conclusions

A promising architecture to detect 8 faults of a PV array has been presented. This diagnostic strategy comprises the new feature eigenvector, including three new feature quantities and the GKFCM. The new feature vectors, including the normalized PV voltage, the normalized PV current and the fill factor, are proposed in the paper to discriminate 5 single faults and 3 compound faults with similar characteristics. The simulation and field experiments demonstrate that the proposed new eigenvectors have a good clustering ability and can differentiate 8 fault types in wide-weather conditions. Since the acquired fault datasets are accompanied by external and internal noise, the GKFCM was adopted to cluster the acquired fault datasets. The GKFCM uses the nonlinear kernel function to map the original feature space to the high-dimensional feature space and then clusters the fault datasets distributed in the high-dimensional space. The mapping procedure can highlight the feature differences of the different fault samples and effectively improve the clustering performance of the complex datasets. In the GKFCM, the similarity function in the support vector machine (SVM) is used as the fault classification function. The diagnostic results validate that the designed similarity function can classify the different faults very well. Finally, the simulation and field experiments were designed to verify the feasibility and effectiveness of the proposed algorithm. 

## Figures and Tables

**Table 1:** Key parameters of JW-G2300-MD6660P-1 photovoltaic (PV) Module.

Description	Value
Maximum power ($P_{mpp-ref}$)	230.3 W
Maximum power point voltage in STC ($V_{mpp-ref}$)	31.00 V
Maximum power point current in STC ($I_{mpp-ref}$)	7.430 A
Open circuit voltage in STC ($V_{oc-ref}$)	37.10 V
Short circuit current in STC ($I_{sc-ref}$)	8.050 A
Temp. dependence of $I_{sc}$ (α)	0.04350
Temp. dependence of $V_{oc}$ (β)	−0.3515
Number of cells in series ($N_{ser}$)	60.00

**Table 2:** Eight fault categories description.

Fault Types	Descriptions	Labels
Case 1	no faults	normal
Case 2	one string in open-circuit condition	open1
Case 3	two string in open-circuit condition	open2
Case 4	one module in short-circuit condition	short1
Case 5	two modules distributed in one string in short-circuit condition	short2
Case 6	two modules distributed in two different strings respectively in short-circuit condition	s1s1
Case 7	one module in short-circuit condition and another string in open-circuit condition	s1o1
Case 8	two modules distributed in two different strings respectively in short-circuit condition and the other string is in open-circuit condition	s1s1o1

**Table 3:** The range of environmental parameters in simulation experiment.

Datasets	Irradiance/W·m^−2^	Temperature/°C
Training datasets	200	0–20
Testing datasets	450–900	10–20

**Table 4:** Center-point coordinates of 8 clusterings in the reference fault datasets.

Fault Types	Labels	Coordinates
case 1	normal	(0.8691, 0.9268, 0.8053)
case 2	open1	(0.8685, 0.6178, 0.8049)
case 3	open2	(0.8682, 0.3089, 0.8044)
case 4	short1	(0.6843, 0.9316, 0.8020)
case 5	short2	(0.4569, 0.9368, 0.8071)
case 6	s1s1	(0.6639, 0.9292, 0.8061)
case 7	s1o1	(0.6718, 0.6200, 0.8048)
case 8	s1s1o1	(0.6524, 0.6174, 0.8052)

**Table 5:** Eight fault identification accuracy in testing phase of simulation experiment.

Fault Types	Sample Number for Identification	Identified Sample Number
normal	110	110
open1	110	110
open2	110	110
short1	110	110
short2	110	110
s1s1	110	110
s1o1	110	110
s1s1o1	110	110

**Table 6:** Key specifications of GSP-240 PV Module.

Description	Value
Maximum power ($P_{mpp-ref}$)	240.0 W
Maximum power point voltage in STC ($V_{mpp-ref}$)	37.60 V
Maximum power point current in STC ($I_{mpp-ref}$)	8.540 A
Open circuit voltage in STC ($V_{oc-ref}$)	29.60 V
Short circuit current in STC ($I_{sc-ref}$)	8.110 A
Temp. dependence of $I_{sc}$ (α)	0.1750
Temp. dependence of $V_{oc}$ (β)	−0.4882
Number of cells in series ($N_{ser}$)	60.00

**Table 7:** Eight fault and unknown fault identification accuracy in testing phase of the field experiment.

Fault Types	Sample Number for Identification	Identified Sample Number
normal	120	118
open1	120	119
open2	120	119
short1	120	72
short2	120	116
s1s1	120	67
s1o1	120	79
s1s1o1	120	66
unknown fault	120	0

**Table 8:** Eight fault and an unknown fault identification accuracy in testing phase of the field experiment.

Fault Types	Sample Number for Identification	Identified Sample Number
normal	120	120
open1	120	117
open2	120	119
short1	120	109
short2	120	117
s1s1	120	101
s1o1	120	117
s1s1o1	120	102
unknown fault	120	0

**Table 9:** Eight faults and unknown fault identification accuracy in testing phase of the field experiment.

Fault Types	Sample Number for Identification	Identified Sample Number
normal	120	117
open1	120	117
open2	120	119
short1	120	73
short2	120	112
s1s1	120	55
s1o1	120	58
s1s1o1	120	96
unknown fault	120	all identified as normal

**Table 10:** Eight faults and the unknown fault identification accuracy in testing phase of the field experiment.

Fault Types	Sample Number for Identification	Identified Sample Number
normal	120	118
open1	120	117
open2	120	119
short1	120	115
short2	120	116
s1s1	120	104
s1o1	120	117
s1s1o1	120	101
unknown fault	120	all identified as normal
